# Gel-immersion biliary cannulation during a balloon-assisted endoscope-guided endoscopic retrograde cholangiopancreatography

**DOI:** 10.1055/a-2575-3533

**Published:** 2025-04-28

**Authors:** Tesshin Ban, Yoshimasa Kubota, Shun Sasoh, Tomoaki Ando, Takashi Joh

**Affiliations:** 1 36884 Department of Gastroenterology and Hepatology, Gamagori City Hospital, Gamagori, Japan

In patients with surgically altered anatomy, a balloon-assisted endoscope-guided endoscopic retrograde cholangiopancreatography (BAE-ERCP), a relatively challenging procedure, is performed for pancreaticobiliary interventions 1 . Gel-immersion endoscopy is a practical aid to help maintain endoscopic views even during ERCP 2 3 . Herein, we present a gel-immersion biliary cannulation used for BAE-ERCP.

An 80-year-old man with a history of Billroth II reconstruction presented with obstructive jaundice due to stage IV pancreatic cancer. Magnetic resonance imaging revealed an aggressive tumor with invasion of the common bile duct into the left Glissonʼs capsule ( Video 1 ). Transpapillary right-lobar drainage using BAE-ERCP was performed as the only method for endoscopic drainage. First, a balloon-assisted endoscope (EI-580BT; Fujifilm, Tokyo, Japan) with a transparent hood was advanced through the afferent loop up to the papilla under CO _2_ insufﬂation. Next, the hidden papillaʼs orifice was approached for wire-guided biliary cannulation; however, the endoscopic view gradually collapsed due to compression by the nearby tumor, even with the underwater method ( Fig. 1 **a, b** ). The air in the afferent loop was then replaced with a transparent gel (Viscoclear; Otsuka Pharmaceutical, Tokyo, Japan). The gel immersion provided an excellent view to help access the papillaʼs orifice by providing sufficient luminal inflation ( Fig. 1 **c, d** ). The biliary duct was accessed ( Fig. 2 ), followed by the successful indwelling of uncovered and covered metallic stents without gel-related adverse events ( Video 1 ).


Biliary cannulation in balloon-assisted endoscope-guided endoscopic retrograde cholangiopancreatography: comparison of CO _2_ insufﬂation/underwater method vs. gel immersion.
Video 1

**Fig. 1:**
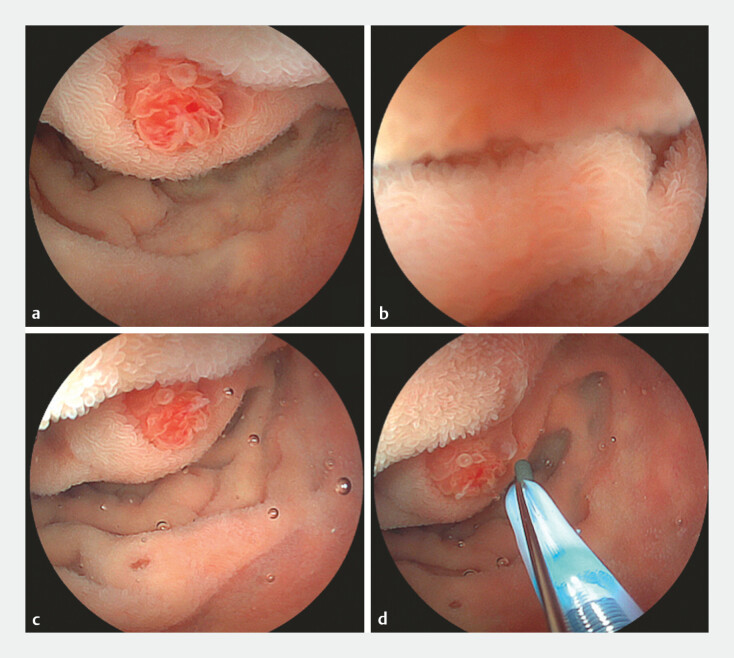
Endoscopic view of the papilla under water and gel immersion. **a, b** Water immersion temporally inflates the duodenal lumen. However, the lumen gradually collapses due to compression by the tumor surrounding the biliary system, which precludes access to the papillaʼs orifice. **c, d** Gel immersion keeps sufficient luminal inflation and provides an excellent view during access to the papillaʼs orifice. Floating microbubbles are recognized in the views.

**Fig. 2:**
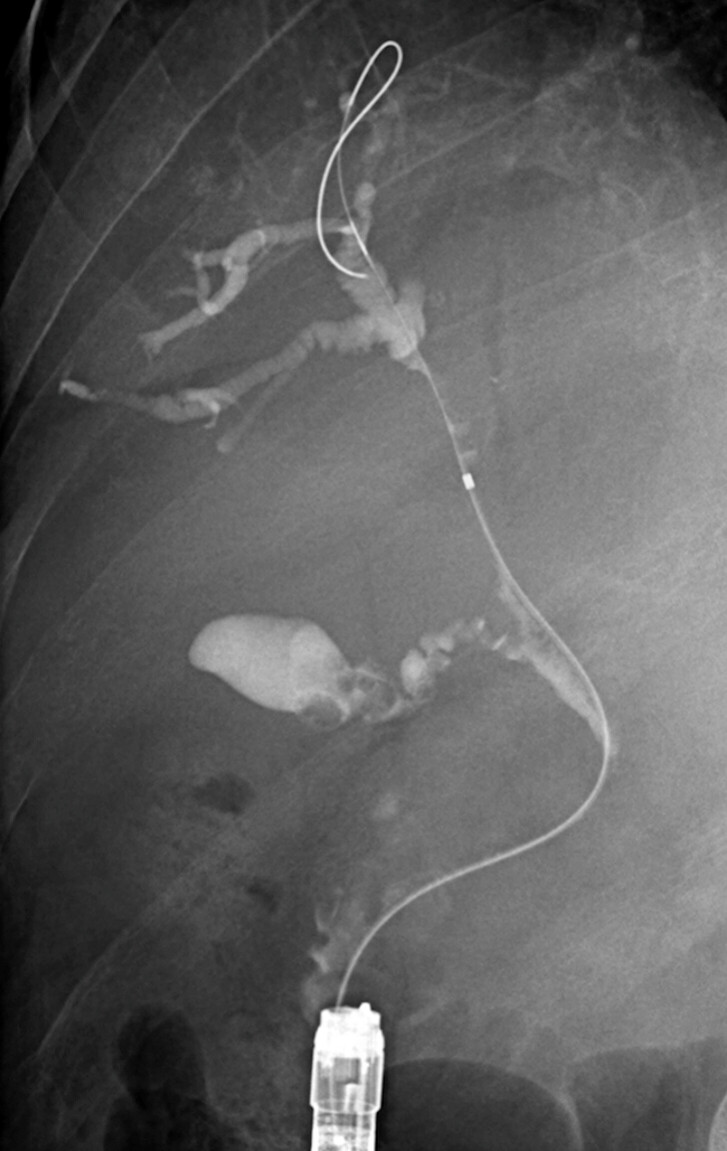
Achieved cholangiography after biliary cannulation. Cholangiography reveals whole-length obstruction of the common bile duct, and only the right bile duct remains intact.

The CO _2_ insufﬂation or water immersion during BAE-ERCP is recommended to avoid the fatal adverse event of systemic gas embolism; however, this adverse event may still occur even with CO _2_ insufflation 4 5 . The CO _2_ or water quickly leaked regardless of the occluded balloon. In contrast, the high-viscosity gel stayed in the enclosed lumen longer. Gel immersion maintained an excellent endoscopic view and potentially minimized gas insufﬂation for biliary cannulation during the BAE-ERCP.

Endoscopy_UCTN_Code_TTT_1AR_2AC

## Notes

Endoscopy E-Videos https://eref.thieme.de/e-videos
*E-Videos* is an open access online section of the journal *Endoscopy* , reporting on interesting cases and new techniques in gastroenterological endoscopy. All papers include a high-quality video and are published with a Creative Commons CC-BY license. Endoscopy E-Videos qualify for HINARI discounts and waivers and eligibility is automatically checked during the submission process. We grant 100% waivers to articles whose corresponding authors are based in Group A countries and 50% waivers to those who are based in Group B countries as classified by Research4Life (see: https://www.research4life.org/access/eligibility/ ). This section has its own submission website at https://mc.manuscriptcentral.com/e-videos .
